# CD147-CAR-NK cell therapy shows minimal toxicities in human CD147 transgenic mouse model with solid tumors

**DOI:** 10.1016/j.omton.2025.200957

**Published:** 2025-02-26

**Authors:** Youssef Sabha, Sang Hoon Kim, Hsiang-chi Tseng, Maeve Elizabeth Byrne, Wei-Chung Tsao, Sang Hoon Lee, Zhongren Zhou, Mi-Hyeon Jang, Dongfang Liu

**Affiliations:** 1 Department of Pathology, Immunology and Laboratory Medicine, Rutgers University New Jersey Medical School, 180 South Orange Avenue, Newark, NJ 07103, USA; 2 Department of Neurosurgery, Robert Wood Johnson Medical School, The State University of New Jersey, 661 Hoes Lane West, Piscataway, NJ 08854, USA; 3 Department of Pathology and Laboratory Medicine, Robert Wood Johnson Medical School, The State University of New Jersey, 1 Robert Wood Johnson Place, New Brunswick, NJ 08901, USA; 4 Center for Immunity and Inflammation, New Jersey Medical School, Rutgers-The State University of New Jersey, 205 South Orange Avenue, Newark, NJ 07101, USA

**Keywords:** natural killer cell, NK cell, chimeric antigen receptor, CAR, CD147-CAR-NK, hepatocellular carcinoma, HCC, toxicity, solid tumors, neurotoxicity

## Abstract

The toxicity of chimeric antigen receptor-natural killer (CAR-NK) therapy has not been tested in solid tumors, compared with CAR-T therapy side by side. To address this, we investigated the CD147-CAR-NK “on-target/off-tumor” toxicity and neurotoxicity in human CD147-transgenic (hCD147TG) mice with hepatocellular carcinoma (HCC). We first tested the *in vitro* cytotoxicity of CD147-CAR-NK against CD147^+^ tumor and CD147^+^ healthy cells. Both CD147-CAR-NK cells and CD147-IL15-CAR-NK (autocrine expressing interleukin [IL]-15) can kill tumor cells specifically but not CD147^+^ healthy lung and spleen tissue from hCD147TG mice. *In vivo* assays show minimal systemic toxicities against CD147^+^ healthy tissues but 1-week-longer persistence times in tumor than non-tumor tissues. To evaluate neurotoxicity, we compared the expression of ionized calcium-binding adaptor protein 1 (IBA1), glial fibrillary acidic protein (GFAP), and inducible nitric oxide synthase (iNOS) between CD147-CAR-T- and CD147-CAR-NK-treated hCD147TG mice with HCC. Both CD147-CAR-T- and CD147-CAR-NK-treated mice exhibited higher GFAP and IBA1 expression than control groups. CD147-CAR-T-treated mice showed an increase in iNOS compared to the control groups. The behavioral studies testing spatial memory showed that mice treated with CD147-CAR-NK exhibit better memory function than CD147-CAR-T-treated mice. This study provides a deeper understanding of the CD147-CAR-NK systemic toxicities and neurotoxicity of CD147-CAR-NK relative to CD147-CAR-T therapy.

## Graphical abstract

**Figure:**
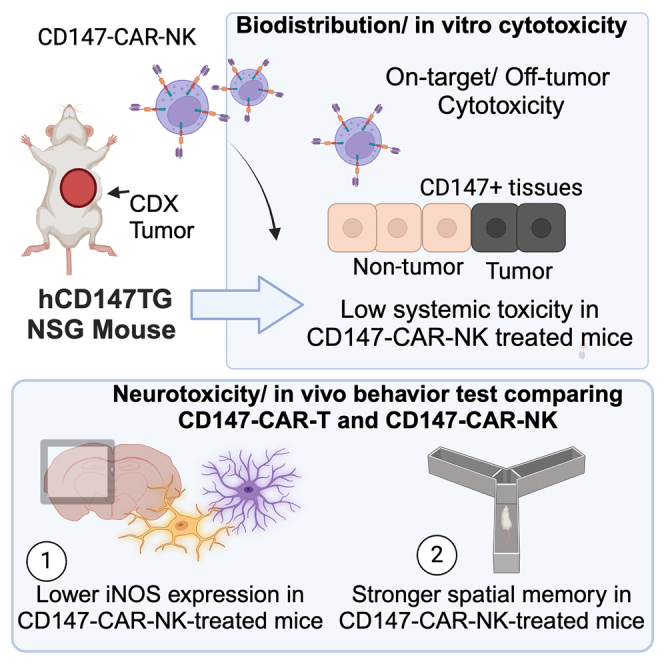

## Teaser

To date, the safety profile of CD147-targeting CAR-NK for solid tumors is unclear. Liu and colleagues present a preclinical evaluation of CD147-CAR-NK therapy in human CD147 transgenic mice with HCC. The authors found CD147-CAR-NK to have minimal toxicities in mice, including less neurotoxicity than CD147-CAR-T.

## Introduction

Chimeric antigen receptor (CAR) therapy shows promising efficacy in treating hematological malignancies, leading to long-term remission in clinical trials for refractory blood cancers.^1^^,^^2^^,^^3^^,^^4^^,^^5^ There are hundreds of clinical trials testing the effectiveness of CAR therapy in the treatment of both solid and blood cancers.^6^^,^^7^ CAR molecules are retrovirally transduced into autologous T cells, natural killer (NK) cells, or macrophages. These genetically modified CAR cells are infused back into patients to treat tumors that are immune evasive to the pre-existing immune system.^8^ CAR-T cells targeting the CD19 antigen are the first cell-based gene therapy to receive FDA approval for treating B cell lymphoma.^1^ There is still an overwhelming need for CAR therapy that targets solid tumors safely and precisely. CAR-T therapies are currently developed to target several tumor-associated antigens (e.g., GD2 and mesothelin) with ongoing evaluation for preclinical efficacy.^9^^,^^10^ CAR-NK and CAR-T therapies continue to show limited efficacy for treatment in many other solid malignancies. A major hurdle for CAR therapy treatment of solid tumors is overcoming the immunosuppressive tumor microenvironment and “on-target/off-tumor” and “on-target/off-tissue” toxicity based on a limited number of identified tumor-specific antigens.^11^^,^^12^

While CD19-CAR-T therapy effectively controls tumor cells in patients with acute lymphoblastic leukemia (ALL), depending on the CAR constructs and manufacturing, there are severe toxicities in a subset of patients, such as cytokine release syndrome (CRS) and immune effector cell-associated neurotoxicity syndrome (ICANS).^13^^,^^14^^,^^15^ CRS induces tissue damage via blood-brain barrier (BBB) disruption and progressive immune cell activation, which can lead to severe neurotoxicity.^14^^,^^16^^,^^17^^,^^18^

Unlike T cells, NK cells persist for a relatively shorter duration in the blood and cause less severe toxicities, suggesting a safer immunotherapy option for patients.^2^^,^^19^ Thus, CAR-NK therapy has “off-the-shelf” manufacturing potentials, unlike its CAR-T counterpart.^20^

Additionally, CAR-NK expansion *ex vivo* has been developed using feeder cell expansion systems, which was a significant limitation to developing CAR-NK for clinical use.^21^ The next steps for CAR-NK therapy are to expand the therapeutic pipeline for solid tumors and optimize the durable response rate to achieve a greater overall objective response of patients, as well as management of CAR-NK toxicities. We developed a CD147 (basigin)-targeting CAR-NK therapy that shows suppression of tumor growth in a cell-line-derived xenograft model of hepatocellular carcinoma (HCC).^22^ Currently, three CD147-CAR-T clinical trials are underway, while the CD147-CAR-NK is still in preclinical studies.^23^ However, the toxicities of CD147-CAR therapies are poorly understood.

Due to the different sequences of CD147 between mice and humans, our published work could not assess systemic off-target toxicities in SARS-CoV-2 infection.^24^ Although the CD147-targeting CAR shows promise for treating solid tumor models (e.g., HCC) and different levels of glycosylation on CD147 between tumors (highly glycosylated on tumors) and healthy tissues (low-level, less-glycosylated CD147 expression in healthy cells), human CD147 (hCD147) is expressed in several tissues, such as the brain and lungs, suggesting potential toxicities.^24^^,^^25^ Thus, we developed a mouse model that expresses hCD147 (hCD147-transgenic [hCD147TG]) in NSG (NOD.Cg-PrkdcSCID Il2rgtm1Wjl/SzJ) mice to evaluate efficacies in a previous study.^24^ This model allows us to evaluate the efficacies and toxicities of CD147-CAR-NK on solid cancers.

This study compared the systemic toxicities and neurotoxicity associated with the CD147-CAR-NK and CD147-CAR-T treatments side by side in the hCD147TG mice with HCC. We demonstrate that CD147-CAR-NK cells have less neurotoxicity than CD147-CAR-T therapy in an hCD147TG mouse model with HCC. To our knowledge, our study is the first to assess the preclinical neurotoxicity and biodistribution of CAR-NK therapy in an hCD147TG mouse model with HCC. This work calls for clinical trials of CD147-CAR-NK cells for cancer treatment.

## Results

### CD147-CAR-NK cells exhibit minimal on-target/off-tumor toxicities on healthy tissues isolated from tumor-bearing hCD147TG mice

**Figure 1:**
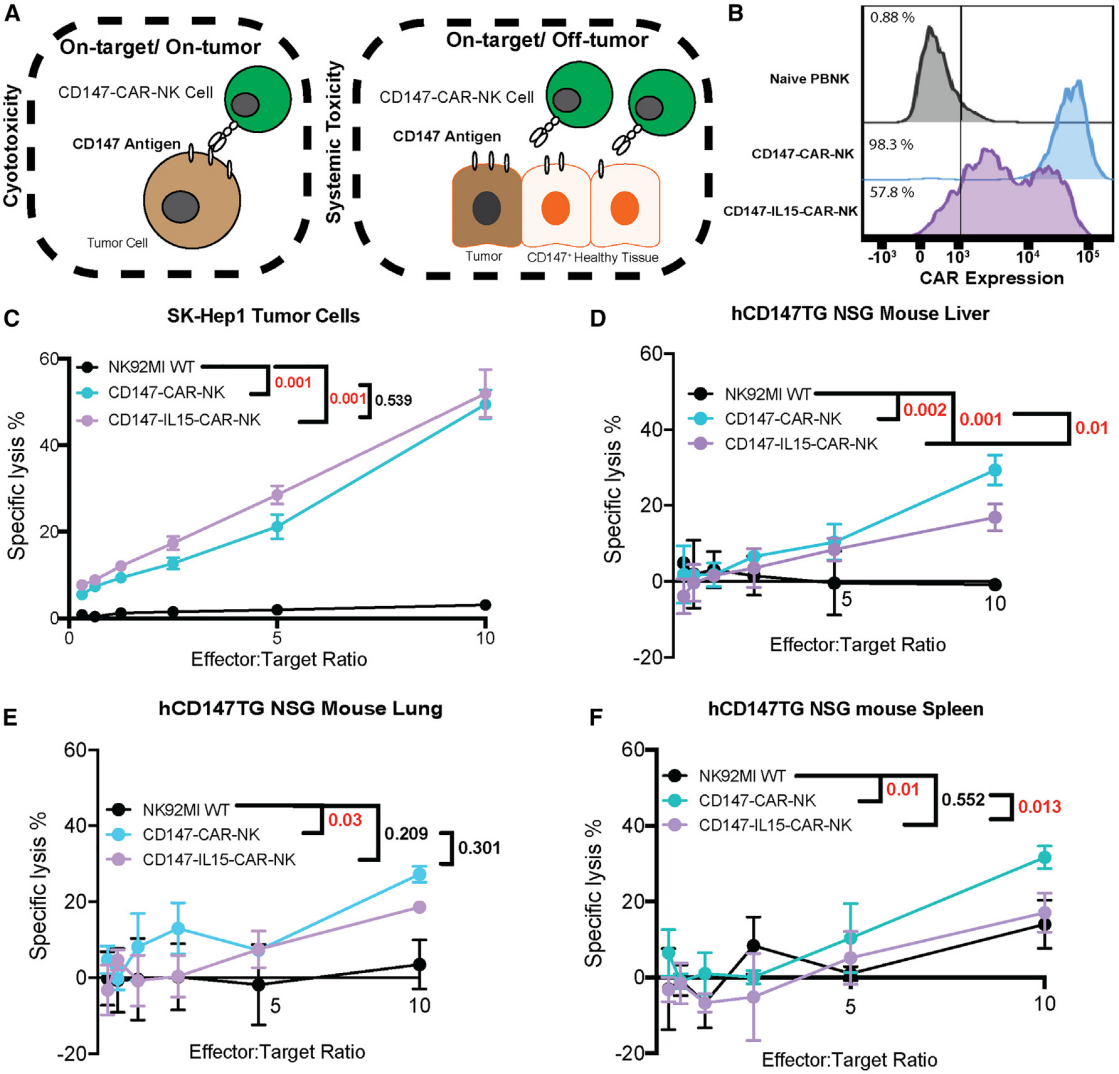
CD147-CAR-NK therapy shows better *in vitro* cytotoxicity against tumor cells than of healthy hCD147TG mouse tissues (A) Schematic outlining the definition of on-target/off-tumor cytotoxicity evaluated in the assays. (B) Flow cytometry histogram showing the percentage of CAR-positive peripheral blood feeder-expanded NK cells transduced with either CD147-CAR or CD147-IL15-CAR. (C) Percentage of specific lysis of engrafted SK-Hep1 tumor cells comparing CD147-IL15-CAR-NK to CD147-CAR-NK with NK92MI as a negative control during a 4-h incubation period. (D) Percentage of specific lysis of isolated hCD147TG mouse liver cells comparing CD147-IL15-CAR-NK to CD147-CAR-NK with NK92MI as a negative control during a 4-h incubation period. (E) Percentage of specific lysis of isolated hCD147TG mouse lung cells comparing CD147-IL15-CAR-NK to CD147-CAR-NK with NK92MI as a negative control during a 4-h incubation period. (F) Percentage of specific lysis of isolated hCD147TG mouse spleen cells comparing CD147-IL15-CAR-NK to CD147-CAR-NK with NK92MI as a negative control during a 4-h incubation period. Values are shown as means (SD) per effector:target ratio. *p* value was calculated using a t test performed on the means between the indicated variables at a 10:1 effector:target ratio.

Survival and weight changes of mice cannot fully evaluate physiological toxicities by CAR-NK cells. We isolated transgenic mouse lung, liver, spleen, and SK-Hep1-engrafted tumor cells from hCD147TG NSG mice for *in vitro* killing assays to test on-target/off-tumor toxicity and on-target/on-tumor cytotoxicity mediated by CD147-CAR-NK cells (Figure 1A). Interleukin (IL)-15 cassettes added to CAR constructs show improved persistence and efficacy in preclinical studies.^26^ Therefore, we also transduced a CD147-IL15-CAR construct with an autocrine expressing IL-15 domain into NK cells (henceforth CD147-IL15-CAR-NK) (Figure S1). Peripheral-blood-derived and feeder-expanded NK cells from healthy donors were transduced with both constructs (Figure 1B). Cells harvested from the SK-Hep1-engrafted tumor, mouse liver, mouse lung, and mouse spleen from hCD147TG mice were co-cultured with either CD147-CAR-NK or CD147-IL15-CAR-NK genetically modified cells from different donors. NK92MI cells, previously shown to not induce cytotoxicity in the harvested tissues, were used as a negative control.^22^ CD147-CAR-NK and CD147-IL15-CAR-NK cells show more than 50% specific killing against SK-Hep1 tumor cells (Figure 1C). The CAR-NK cells show less than 30% specific lysis of the hCD147TG mouse liver and lung cells, with minor differences between CD147-CAR-NK and CD147-IL15-CAR-NK effector cells (Figures 1D and 1E). The CD147-CAR-NK cells show less than 40% specific lysis of hCD147TG mouse spleen cells and non-significant cytotoxicity by CD147-IL15-CAR-NK cells (Figure 1F). The killing of tumor cells is more significant than that of non-tumor tissues, suggesting lower on-target/off-tumor than on-target/on-tumor toxicities. In summary, CD147-CAR-NK exhibits precise on-tumor cytotoxicity with lower on-target/off-tumor killing, suggesting low toxicity to healthy tissues that express CD147.

### CD147-CAR-NK therapy shows minimal systemic toxicities on tumor-bearing hCD147TG mice

**Figure 2:**
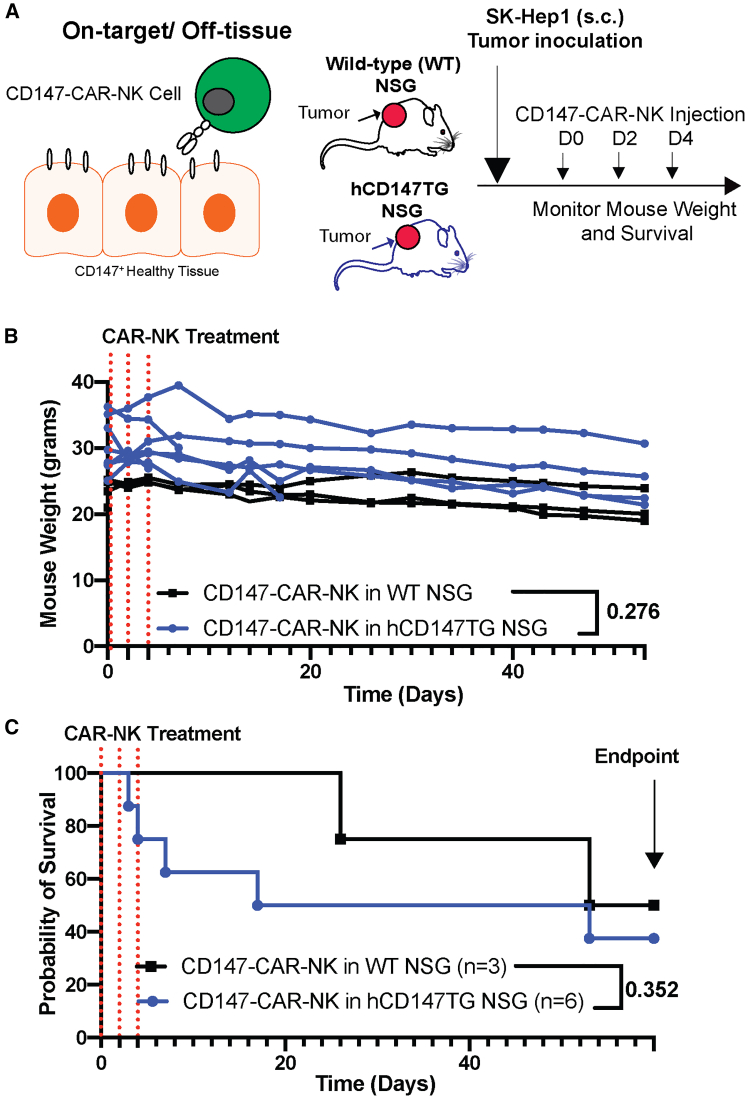
CD147-CAR-NK therapy exhibits non-significant systemic effects on tumor-bearing hCD147TG mouse weight and survival (A) Experimental design to evaluate on-target/off-tissue toxicity by mouse survival and weight analysis. (B) Mouse weight of individual mice collected twice weekly is recorded with WT NSG mice (*n* = 3) or hCD147TG mice (*n* = 6). *p* values were calculated using a two-way ANOVA comparing mouse weight between wild-type and hCD147TG mice. (C) Survival of either wild-type or hCD147TG NSG mice treated with CD147-CAR-NK is recorded. *p* value was calculated using a log rank comparison Mantel-Cox test.

Next, we aim to evaluate systemic on-target/off-tissue toxicities of the therapy in hCD147TG mice. To evaluate the systemic toxicity of CD147-CAR-NK therapy in a cell-line-derived xenograft (CDX) HCC tumor model, we treated tumor-bearing hCD147TG NSG and tumor-bearing wild-type (WT) NSG mice with three doses of CD147-CAR-NK therapy at different time points (Figure 2A). Transduced peripheral-blood-derived NK cells from healthy donors contain the most clinically relevant phenotypes; therefore, we tested a CD147-CAR construct that was used in a previous study.^22^ There were no significant differences in the body weight of WT NSG and hCD147TG mice treated with CD147-CAR-NK therapy for 53 days (Figure 2B). The hCD147TG mice showed no significant differences in survival after treatment throughout the 53-day longitudinal experiment compared to WT NSG mice (Figure 2C). Comparing the two mouse backgrounds, the lack of reduced survival and weight decreases suggests low on-target/off-tissue systemic toxicities in mice treated with CD147-CAR-NK cell constructs. In conclusion, CD147-CAR-NK showed low systemic toxicities to the tumor-bearing hCD147TG mice.

### Both CD147-CAR-NK and CD147-IL15-CAR-NK cells exhibit 1-week-longer persistence in the tumor tissues than in healthy liver, lung, and peripheral blood in tumor-bearing hCD147TG mice

**Figure 3:**
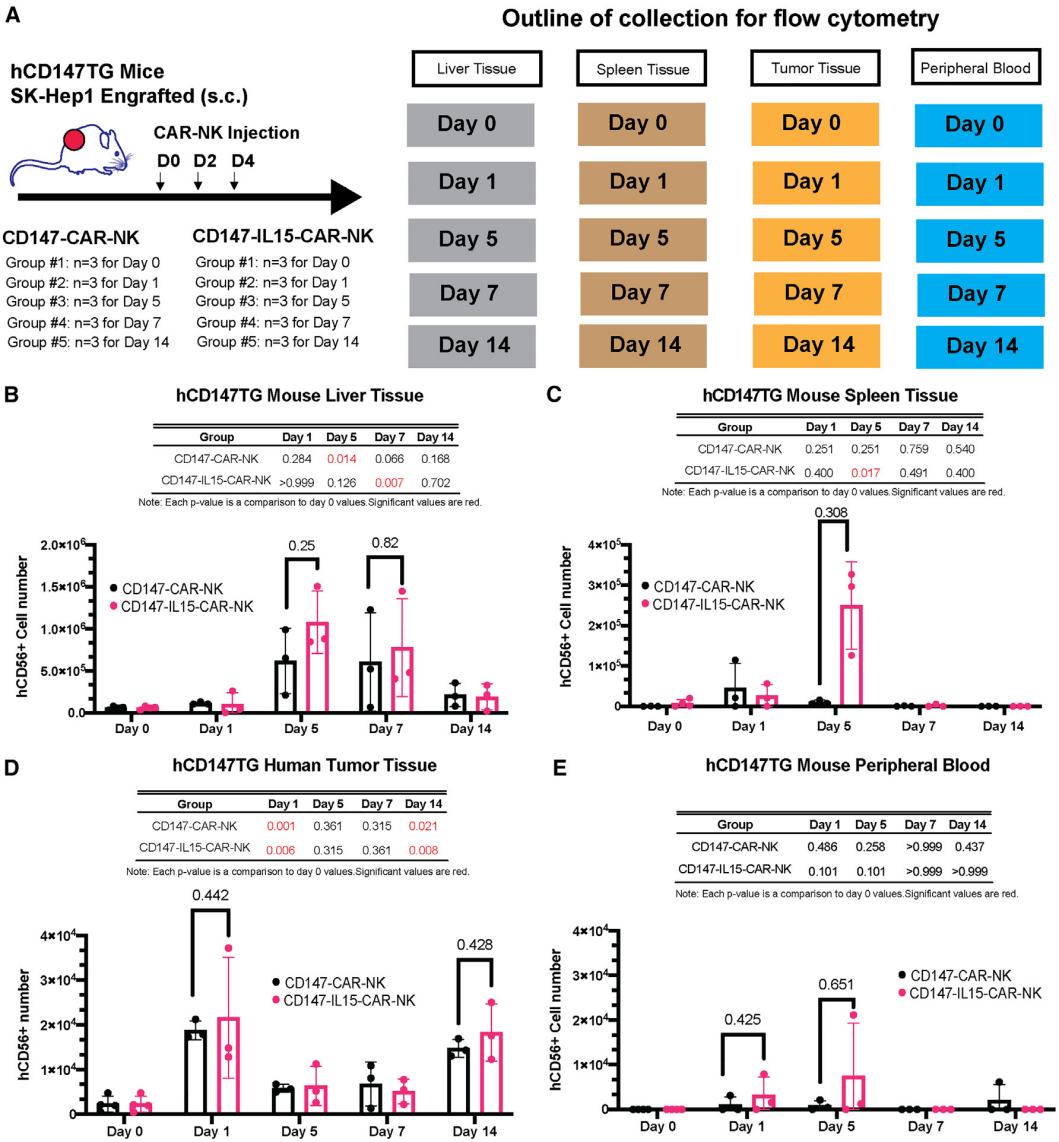
CD147-CAR-NK therapy persists longer in tumor tissue than in hCD147TG non-tumor tissues (A) Schematic outlining experimental design and workflow. Human CD56^+^ (hCD56^+^) cells of both CD147-IL15-CAR and CD147-CAR backgrounds were counted using flow cytometry at day of harvest. (B) Mean of absolute hCD56^+^ cell count from harvested hCD147TG mouse liver tissue. (C) Mean of absolute hCD56^+^ cell count from harvested hCD147TG mouse spleen tissue. (D) Mean of absolute hCD56^+^ cell count from harvested SK-Hep1 tumor tissue. (E) Mean of absolute hCD56^+^ cell count from harvested hCD147TG mouse peripheral blood on indicated days. All values are shown as means (SD). *p* values were calculated using a one-way ANOVA analysis on the means. *p* values are displayed with associated bar chart.

Next, we aim to evaluate CAR cell biodistribution to investigate on-target/off-tissue persistence. The *in vitro* assay does not entirely address *in vivo* biodistribution or off-tissue toxicities. CAR-T/CAR-NK persistence in the tissues is directly correlated with CAR efficacy.^27^^,^^28^^,^^29^ While there is published data suggesting the short persistence of NK cells, we aim to evaluate the *in vivo* biodistribution of our CD147-CAR-NK therapy in an hCD147TG mouse model.^30^ The distribution and persistence time of CD147-CAR-NK after an intravenous (i.v.) injection are unclear. Transgenic tumor-bearing mice were split into 10 cohorts and treated with either CD147-CAR-NK or CD147-IL15-CAR-NK therapy, and tissues were harvested on days 0, 1, 5, 7, and 14 (Figure 3A). hCD56^+^ NK cells were gated in harvested tissues (Figure S2). Liver tissue in the hCD147TG mouse shows positive expression of the hCD56^+^ cells at day 7 post-injection, with reductions by day 14 post-injection (Figure 3B). There were non-significant differences in biodistribution between hCD56^+^ cells with and without the autocrine IL-15 (Figure 3B). hCD56^+^ cells from CD147-IL15-CAR-NK-treated hCD147TG mice were distributed in the spleen at 1 day post-injection of CAR for up to 5 days and low amounts of hCD56^+^ cells by day 7 (Figure 3C). Both CD147-CAR-NK and CD147-IL15-CAR-NK therapies exhibit distribution in the tumor for up to 14 days, with the highest absolute expression 1 day post-CAR injection (Figure 3D). Although the CAR-NK cells were injected i.v., non-significant hCD56^+^ cells persisted in the mouse blood after injection (Figure 3E). Unlike CAR-T therapies, which persist in peripheral blood and tissues for several weeks or months in mice and other preclinical models, the CAR-NK data showed a short persistence of cells in non-tumor tissues, with the longest persistence in tumor tissues.^5^^,^^31^ The biodistribution of CD147-CAR-NK showed persistence in tumor tissues 1 week longer than healthy hCD147TG tissues, which can explain the low on-target/off-tissue toxicities in CD147-CAR-NK therapy.

### CD147-CAR-NK therapy exhibits less neuroinflammation than CD147-CAR-T treatment of tumor-bearing hCD147TG mice

**Figure 4:**
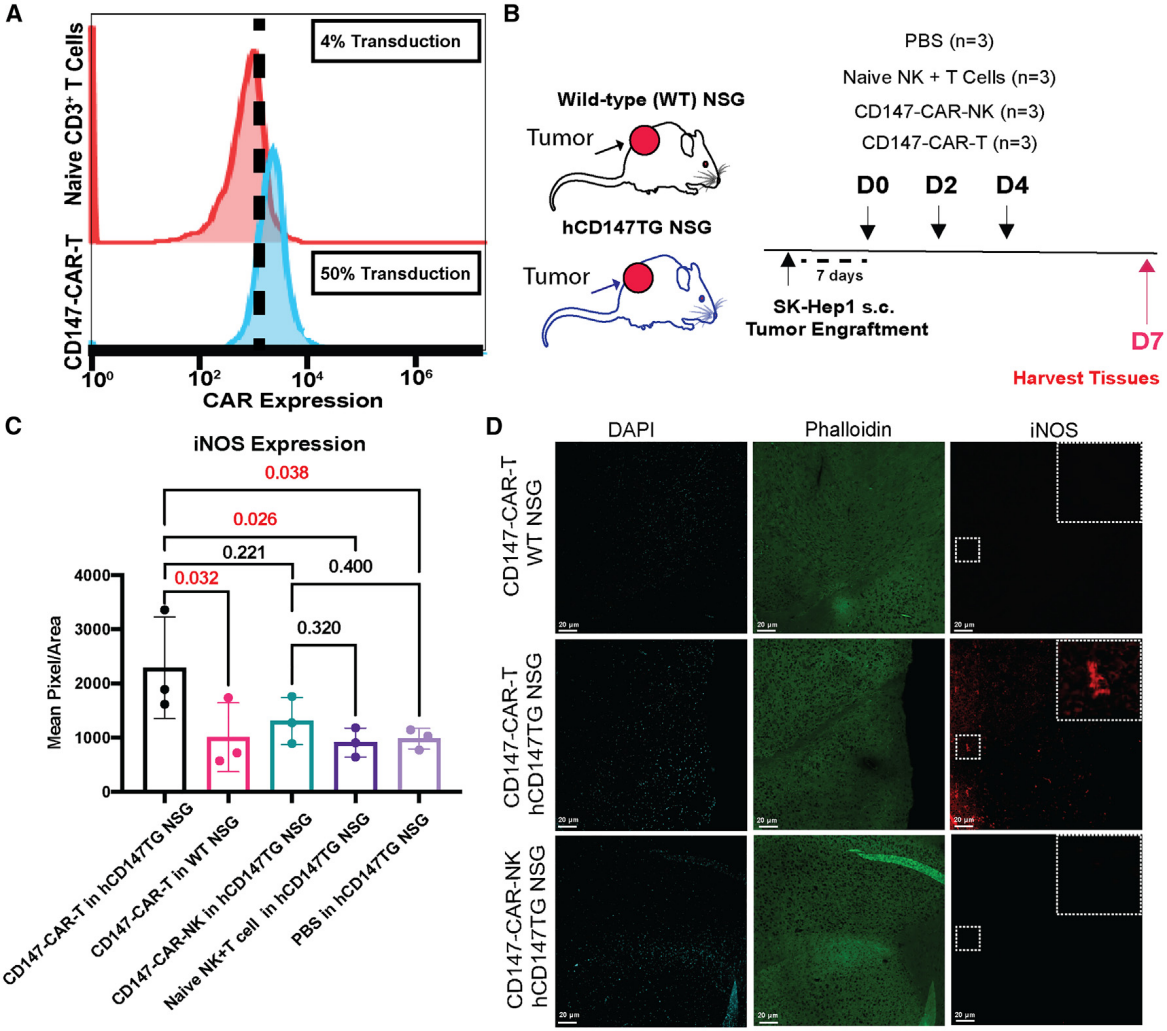
CD147-CAR-NK therapy induces less neuroinflammation than CD147-CAR-T therapy in hCD147TG mice inoculated with SK-Hep1 cells (A) Flow cytometry histogram of CD3^+^ T cells transduced with the CD147-CAR construct. (B) Schematic showing hCD147TG and wild-type NSG mice treatment with three doses of either CD147-CAR-T or CD147-CAR-NK, and then brain tissue was harvested at day 7 after the first CAR dose. (C) Mean pixel/area of iNOS expression in harvested brain tissues comparing CD147-CAR-NK- and CD147-CAR-T-treated hCD147TG mice to naive NK+T cell-injected hCD147TG mice. (D) Representative confocal microscopy of randomized brain field expressions of DAPI, phalloidin, and iNOS of CD147-CAR-NK- and CD147-CAR-T-treated hCD147TG mice and CD147-CAR-T-treated NSG mice as a negative control. Scale bars = 20 µm. Values were shown as means (SD). *p* value was calculated using one-way ANOVA on means.

Several studies have linked CAR-T therapy persistence to neurotoxicity, whereby T cells induce a significant activating effect on other immune cells over a long period, leading to cytokine storm and severe tissue damage.^32^^,^^33^^,^^34^ Notably, CD147 is also expressed in brain tissues.^24^ However, it remains unclear whether the brain is affected by “off-tissue” cytotoxicity. Thus, we aimed to evaluate if the brain is affected by this short-term persistence. Since CAR-T is known to cause neurotoxicity, we transduced the CD147-CAR into expanded CD56^−^CD3^+^ T cells as a positive control (Figure 4A). T cell development and persistence are not less dependent on IL-15 compared to NK cells; therefore, for a precise comparison to CAR-T therapy, we used the CD147-CAR construct for the neurotoxicity study. WT and hCD147TG NSG mice inoculated with SK-Hep1 cells were injected with three doses of either CD147-CAR-NK or CD147-CAR-T (5 × 10^6^ cells per dose). Brains were harvested from the mice 7 days after the first injection, and cortex regions were evaluated for neuroinflammation (Figure 4B). Inducible nitric oxide synthase (iNOS; a marker for systemic neuroinflammation) expression is linked to endoplasmic reticulum stress and neuroinflammation as a measure of tissue neuroinflammation.^35^ As expected, there was significantly higher iNOS signaling in CD147-CAR-T-treated hCD147TG NSG mice than in CD147-CAR-T-treated WT mice. Importantly, there is no significant difference between the CD147-CAR-NK iNOS signal and PBS-treated and untransduced (naive) NK+T-treated hCD147TG mouse groups (Figure 4C). Upon imaging the brains, we found the iNOS signal to be homogeneous in the brain tissues, with the CAR-T having the highest signal in the hCD147TG mice (Figures 4D and S3). In conclusion, CD147-CAR-T-treated hCD147TG mice exhibited higher levels of neuroinflammation than CD147-CAR-NK-treated mice, although the mechanism of this phenomenon is unclear.

### CD147-CAR-NK-treated hCD147TG mice with HCC show better spatial memory function than CD147-CAR-T-treated hCD147TG mice with HCC

**Figure 5:**
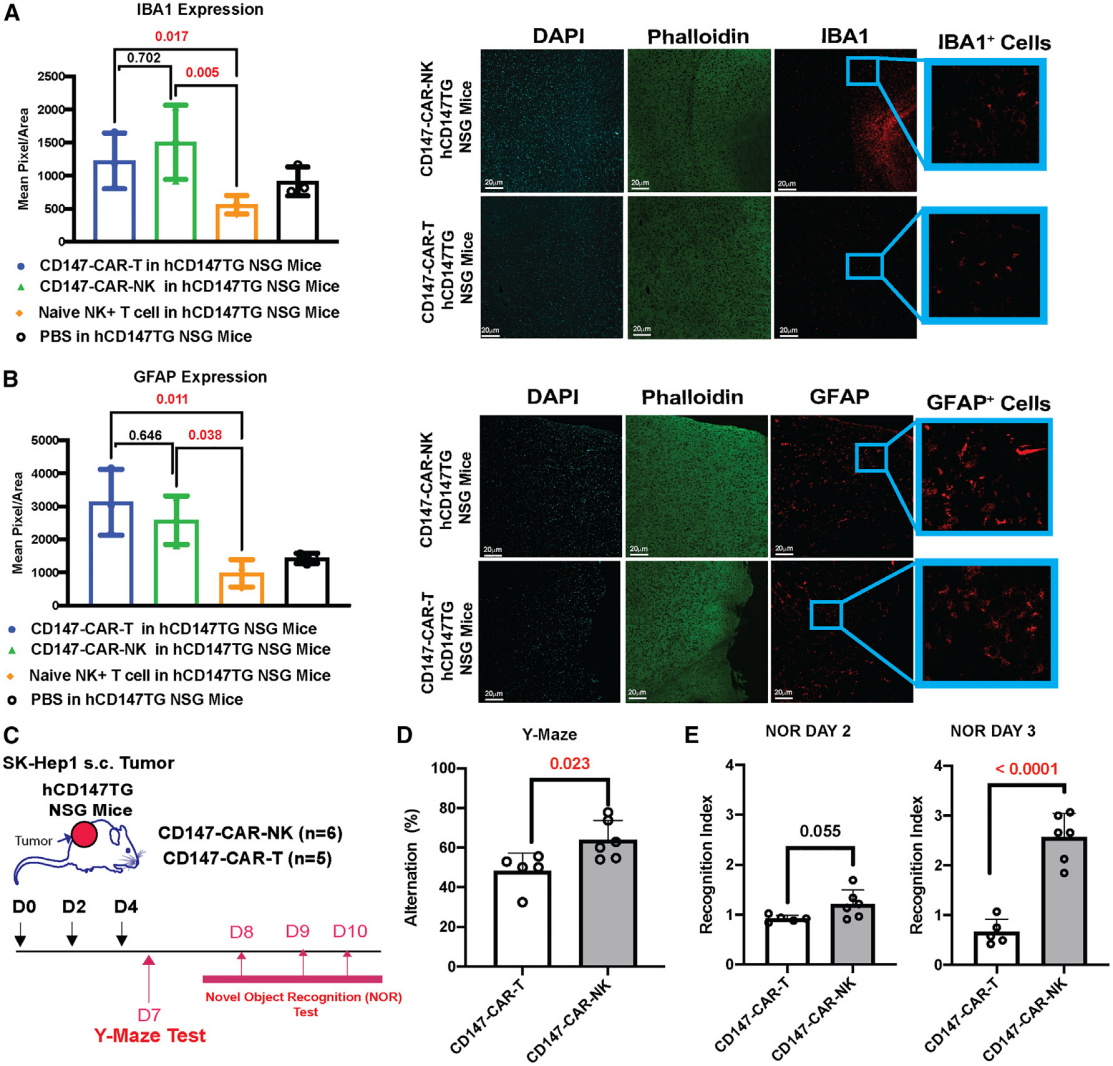
CD147-CAR-NK-treated hCD147TG mice show better spatial memory function than CD147-CAR-T-treated hCD147TG mice with similar immune cell activation profiles (A) Mean pixel/area of IBA1 expression in harvested brain tissues comparing CD147-CAR-NK- and CD147-CAR-T-treated hCD147TG mice to naive NK+T cells and PBS-injected hCD147TG mice. On the right, representative confocal microscopy of randomized brain fields shows the expression of DAPI, phalloidin, and IBA1. (B) Mean pixel/area of GFAP expression in harvested brain tissues comparing CD147-CAR-NK- and CD147-CAR-T-treated hCD147TG mice inoculated with SK-Hep1 cells to naive NK+T-injected hCD147TG mice. On the right, representative confocal microscopy of randomized brain fields shows the expression of DAPI, phalloidin, and GFAP. Scale bar = 20 µm. (C) Schematic of behavioral study timeline in hCD147TG tumor-bearing mice. (D) Mean percentage of alternation (used to quantify the spatial memory) of CAR treatment in hCD147TG mice on day 7 after the first CAR dose. (E) Recognition index on CAR-treated hCD147TG mice NOR days 2 and 3. Values were shown as means (SD), and a one-way ANOVA was performed on means to calculate the *p* values. For behavioral studies, means were compared using an unpaired t test to calculate the *p* values.

Microglial cells and astrocytes are immune cells essential for protecting the brain from disease-induced damage.^18^ Upon damage to the BBB, astrocytes are activated, leading to microglial activation, or vice versa.^17^^,^^36^^,^^37^ We utilized these cell populations as indicators of neuroinflammation caused by the CD147-CAR-NK. Ionized calcium-binding adaptor protein 1 (IBA1; a marker for microglia activation) is a commonly accepted biomarker of microglial activation.^38^ On day 7, we found similar levels of microglial cell activation in both CD147-CAR-T- and CD147-CAR-NK-treated mice, which are significantly higher than those in naive NK+T- and PBS-treated hCD147TG mice groups (Figures 5A and S4). Astrocyte activation is commonly used as a biomarker for damage to the BBB.^39^ Glial fibrillary acidic protein (GFAP; a marker for astrocyte activation) is a widely studied marker of astrocytes.^40^ Upon harvesting, we found similar GFAP expression in the cortex between CD147-CAR-T- and CD147-CAR-NK-treated mice (Figure 5B). The CAR-treated hCD147TG tumor-bearing mice had significantly higher GFAP activation than the naive NK+T and PBS-treated tumor-bearing mouse groups (Figures 5B and S5). We performed behavior assays to assess spatial memory to further evaluate the impact of the physiological and neurological changes. The Y-maze and novel object recognition (NOR) assays are used to determine the spatial and recognition memory of mice, respectively.^41^^,^^42^ Behavioral assays were performed on day 7 after the initial CAR dose in the hCD147TG mice (Figure 5C). The mice treated with CD147-CAR-NK exhibit a significant increase in spontaneous alternation changes compared to CD147-CAR-T-treated mice in the Y-maze test, suggesting better spatial memory function with CD147-CAR-NK treatment (Figure 5D). In addition, our NOR results showed no significant differences in the recognition index exploring the two familiar objects between CD147-CAR-T- and CD147-CAR-NK-treated mice on day 2, suggesting no preference for location exploration. However, on test day 3, when a familiar object was replaced with a novel object, CD147-CAR-NK-treated mice displayed a higher recognition index exploring the novel object relative to CD147-CAR-T-treated mice, indicating better spatial memory function in CD147-CAR-NK-treated mice (Figure 5E). While the histological evaluation of CAR-treated hCD147TG mouse brains shows no observable differences in immune cell infiltration, microglial and astrocyte activation suggests a potential mechanism for iNOS signaling (Figure S6). Overall, the positive neuroimmune cell activation (e.g., astrocytes and microglia) suggests neurotoxicity from CD147-CAR-NK and CD147-CAR-T via potential off-target effects from the CAR therapy, although CD147-CAR-T-treated mice had more deficit in spatial memory function compared to CD147-CAR-NK. In conclusion, CD147-CAR-NK-treated hCD147TG mice show better spatial memory function and less neuroinflammation than CD147-CAR-T-treated hCD147TG mice.

## Discussion

Immunotherapies, such as CAR therapies, are increasingly being used to treat many cancer subtypes, with growing interest in treating solid tumors.^20^ This study shows a strategy to evaluate CAR therapy toxicity in the treatment of solid tumors. CD147-CAR-NK therapy shows promising preclinical efficacy in tumor suppression of solid tumors, although CD147 is a controversial tumor-associated antigen due to the low expression of CD147 in healthy tissues.^43^^,^^44^ The first key finding is low on-target/off-tumor cytotoxicity on hCD147TG mouse tissues *in vitro* by CD147-CAR-NK, whereby tumor cells are killed better than non-tumor hCD147TG mouse cells. Secondly, the data presented in this study show a distribution of CD147-CAR-NK for up to 14 days in the tumor. In non-tumor tissues, such as the spleen, liver, and peripheral blood, the percentage of CAR-NK cells decreased dramatically, which is consistent with previous observations.^45^

The critical gap evaluated in this study is the neurotoxicity of the CD147-CAR-NK therapy. CD147 is expressed in brain tissues^24^; therefore, there are potential on-target/off-tissue neurotoxicity concerns. To address this, we evaluated the effects of CD147-CAR-NK and CD147-CAR-T cells on the activation of microglial cells and astrocytes. While astrocytes and microglial cells are equally activated with both CAR therapies, the damaging effects of the CD147-CAR-T therapy were higher, as indicated by increased iNOS expression across the brain tissues. Additionally, we performed Y-maze and NOR behavior tests to evaluate spatial memory and found that CD147-CAR-NK-treated mice had better memory function than CD147-CAR-T-treated mice. The activation of microglia and astrocytes after CD147-CAR-T is similar to that in related studies on CD19-CAR-T.^46^^,^^47^ In conclusion, while CD147-CAR-NK appears to activate microglial cells and astrocytes, the histological changes and effects on memory are less severe than in CD147-CAR-T-treated mice.

This study shows several novelties in the field of CAR-NK treatment of solid tumors. First, we are the first to do a side-by-side comparison between CAR-T and CAR-NK therapies of a solid-tumor antigen (e.g., CD147). Second, we show while the tumor antigen is expressed at low levels in healthy tissues, there is non-significant toxicity to the healthy tissues in hCD147TG mice. The reasons for this could be (1) that the majority of CD147-CAR-NK cells in the tumor-bearing hCD147TG mice penetrate and persist in the tumor more than the non-tumor tissues; (2) the relatively short lifespan of CD147-CAR-NK cells compared to CD147-CAR-T cells *in vivo*; (3) the different forms of CD147 between tumor and healthy tissues; or (4) the different post-translation modification of CD147 between tumor and healthy tissues, for instance, the levels of glycosylation of CD147 between tumor and healthy tissues. There is less extensive glycosylation of CD147 in healthy tissues.^48^ Finally, we show an accessible and integrated multidisciplinary pipeline to evaluate brain neuroinflammation and cell therapy toxicity.

While CD147-CAR-NK therapy shows a promising safety profile compared to CD147-CAR-T, these studies were performed in immunodeficient mice. Immunocompetent mice will accurately recapitulate both the tumor microenvironment and the immune cells. Increased mouse sample sizes for behavioral and survival studies, as well as underlying molecular mechanisms, are needed to understand the full impact of CAR-transduced NK cells in a preclinical setting.

This study introduced a glimpse into CD147-CAR-NK effects on mouse survival, CAR-NK persistence, and neurotoxicity, suggesting a safer profile than the CAR-T counterpart currently in clinical trials, particularly in solid tumors. Importantly, our study revealed new findings on the safety profile for CD147-CAR-NK treatment and potential therapeutic use on solid tumors. Future studies aim at studying CD147-CAR-T and CD147-CAR-NK experiments in immunocompetent mice with larger study groups. These studies would allow us to study CD147-CAR-NK cell therapy’s cytokine storm profile, BBB damage, other immune cell involvement, and its impact on brain tissues and its function. Understanding the on-target/off-tumor and on-target/off-tissue effects of CAR-NK on murine tissues is critical for advancing the clinical development of CAR-NK therapies.

## Methods

### Antibodies and reagents

Allophycocyanin (APC)- or phycoerythrin (PE)-conjugated anti-hCD3 antibody (clone OKT3, BioLegend); PE-Cy7; fluorescein isothiocyanate (FITC)- or Brilliant Violet (BV)510-conjugated anti-hCD56 antibody (clone HCD56, BioLegend); and Alexa Fluor 647 goat anti-human immunoglobulin G (IgG) F(ab′)2 fragment antibody were purchased from Jackson ImmunoResearch (West Grove, PA, USA) as previously described.^22^ DAPI was purchased from BioLegend (San Diego, CA, USA). The primary antibody against Iba1 (#019-19741) was purchased from Fuji Film Wako Pure Chemical Corporation (Osaka, Japan). Primary polyclonal antibody against iNOS (#PA1036) was purchased from Thermo Fisher Scientific (Waltham, MA, USA). Alexa Fluor 488-conjugated primary antibody against phalloidin (#A12379) was purchased from Thermo Fisher Scientific. The primary polyclonal antibody against GFAP (#Z033429-2) was purchased from Agilent Technologies (Santa Clara, CA, USA). The Alexa Fluor 647 secondary antibody against rabbit IgG (H+L) (#A21244) was purchased from Thermo Fisher Scientific. All primary antibodies used for flow cytometry and immunofluorescence were used at the same final concentration (1:100 dilution) unless otherwise specified. All secondary antibodies for immunofluorescence were used at the same final concentration (1:200).

### Cell lines

SK-Hep1 are liver cancer cells purchased from the American Type Culture Collection (ATCC). The 721.221 mIL21 cell line was previously engineered by the lab and was used to expand CAR-NK cells.^21^

### Generation of CD147-CAR-NK and CD147-CAR-T

Peripheral blood from healthy donors at the New York Blood Center (New York, NY, USA) expanded CD3^+^CD56^−^ T cells and CD3^−^CD56^+^ NK cells. NK cells were expanded using 721.221 mIL21 feeder cells, as previously described.^21^ NK cells were harvested on day 7 of expansion and transduced with CD147-CAR or CD147-IL15-CAR retrovirus in plates coated with RetroNectin. After 48 h, cells were transferred to G-Rex 6 multi-well cell culture plates and maintained in 30 mL of complete NK culture media according to ATCC with 400 U/mL IL-2 and IL-15 (PeproTech). To check the percentage of NK cells and CAR expression, cells were stained for CD3, CD56, and human IgG F(ab′)2 and analyzed by flow cytometry. To expand CD3^+^CD56^−^ T cells, 6 × 10^6^ peripheral blood mononuclear cells were mixed with CD3/CD28 Dynabeads (Thermo Fisher Scientific #11161D) with 400 U/mL IL-2 (PeproTech) for 4 days before use for transduction. T cell medium was changed every 3–4 days, and 2 × 10^7^ cells were kept in each well for continued culture each time. Total cell numbers were counted using acridine orange/propidium iodide.

### ^51^Cr release assay

To evaluate the cytotoxic activity of CAR-NK cells, the standard 4-h ^51^Cr release assay was used as previously described.^22^ Tissues of the mice, specifically the lung, liver, spleen, and tumor, were harvested, minced, and dissociated using a GentleMACS Octo with heater (Miltenyi Biotec) in collagenase IV (Thermo Fisher Scientific). The suspension was filtered through a 70-mm cell strainer before washing. Dissociated target cells were labeled with ^51^Cr at 37°C for 2 h and then resuspended in RPMI media with 10% FBS with IL-2 or IL-15. Then, 2 × 10^5^ target cells were incubated with serially diluted CAR-NK cells at 37°C for 4 h. After centrifugation, the supernatants were collected, and the released ^51^Cr was measured with a gamma counter (Wallac, Turku, Finland). The cytotoxicity is calculated as previously described.^22^

### Animal studies

All animal experiments have been approved by the Rutgers Institutional Animal Care and Use Committee (IACUC, PROTO201800200) and Institutional Review Board (IRB, PROTO20160001036). NSG mice from The Jackson Laboratory (Bar Harbor, ME, USA) were used for all *in vivo* experiments. To establish an HCC cell line xenograft model, both male and female NSG mice (8–10 weeks) were injected subcutaneously with 4 × 10^6^ SK-Hep1 cells in 100 μL of PBS Corning Matrigel matrix in the right flank. When the tumor was palpable, mice were randomly allocated into treatment groups. On day 7 after tumor inoculation, the mice were injected with three doses retro-orbitally (i.v.) with 5 × 10^6^ CD147-CAR-NK cells or 5 × 10^6^ CD147-CAR-NK cells in 100 μL of PBS supplemented with IL-2. Control groups were infused with either parental naive cells or vehicle (PBS). Naive cells were a mixture of NK and T cells (1:1 ratio totaling 5 × 10^6^ cells per dose) expanded from the same donor. Mice were sacrificed 8 days after the first dose of CAR therapy.

### Brain staining and microscopy

Brain tissues were fixed in 4% formalin for 24 h to evaluate the expression of neuroinflammation biomarkers and then transferred into 30% sucrose for 24 h prior to placement into an Optimal Cutting Temperature (OCT) compound block then frozen using an isopropanol/dry ice slush. The brains were sectioned at 12-μm thickness and placed onto glass slides overnight before immunofluorescence staining. Brains were washed with 1× PBS twice, and antigen retrieval was performed using 10 mM sodium citrate. Slides were washed, permeabilized using 1× Triton X-100 (Sigma-Aldrich) for 30 min, blocked for 1h using bovine serum albumin, and then incubated with primary antibody for at least 4 h. Secondary antibody incubation was performed for 1 h, and then glass coverslips were mounted using ProLong Gold Antifade reagent with DAPI (Invitrogen #P36935). Slides were imaged at 10× magnification using the IN-Cell Analyzer 6500HS (GE Healthcare, Seattle, WA, USA).

### Y-maze behavioral test

The Y-maze is a behavior test designed to assess working memory and spatial memory by leveraging the innate tendency of rodents to explore novel, unexplored areas. The Y-maze used in this study consisted of three identical arms (10 × 40 × 15 cm) arranged at 120° angles. Mice were released in the center of the maze and allowed to freely explore the three arms for 5 min, with their behavior recorded on video. The maze was cleaned with 70% ethanol and dried between trials to eliminate any olfactory cues. An arm entry was defined as the mouse’s entire body entering an arm, and the spontaneous alternation score was recorded. The spontaneous alternation score represents instances where the mouse successfully entered all three arms in sequence. The score was calculated using the formula (number of successful sequential visits)/(total number of arm entries − 2).

### NOR behavioral test

The NOR test is a behavior assay utilized to evaluate cognitive function by taking advantage of rodents’ natural proclivity for exploring novel objects and environments. The NOR test was conducted over a period of 3 days. On the first day (habituation phase), mice were placed in the experimental apparatus (40 × 40 × 40 cm) for 10 min to freely explore and acclimate to the environment. On the second day (training phase), two identical objects were placed diagonally within the apparatus, and the mice were allowed to explore both objects for 10 min. On the third day (testing phase), one of the objects was replaced with a novel object, and the mice were again allowed to explore for 10 min. All behavior was recorded on video, and exploration time was measured only when the mice sniffed the objects with their noses. The time spent exploring the novel object relative to the familiar object was used as an index of recognition memory.

### Generation of hCD147TG mice

We developed a transgenic NSG mouse model in which hCD147-specific CAR-T/NK cells could be adoptively transferred into mice whose normal cells and tissues express an hCD147 transgene at heterozygous or homozygous levels as previously described.^24^ The targeted alleles were subsequently sequenced in their entirety by PCR genotyping.

### Graphical abstract and illustrations

The graphical abstract was created using software available at biorender.com. All other figures were made using Adobe Illustrator software.

### Statistical analysis

A statistical analysis evaluating the expression of the neuroinflammation biomarkers was performed using a one-way ANOVA (analysis of variance). Survival curves were analyzed using Kaplan Meier with a log rank (Mantel-Cox) statistical test. The other statistical significance was determined using a two-tailed unpaired Student’s t test or a two-tailed paired Student’s t test. All statistical calculation graphs were generated by GraphPad Prism 5.0 and are shown in the figures. Red *p* values indicate statistical significance.

## Data availability

All relevant data, supplemental information, or information from the corresponding author is available in the article upon reasonable request.

## Acknowledgments

We would like to thank the members of the Liu laboratory for their comments on the manuscript. This work was supported in part by HL125018 (D.L.), AI124769 (D.L.), AI129594 (D.L.), AI130197 (D.L.), CA267368 (D.L.), Rutgers-HealthAdvance funding (NIH REACH program, 10.13039/100000050NHLBI award U01HL150852), and 10.13039/100000005Department of Defense (DOD) HT9425-23-1-0364 (M.-H.J.) and HT9425-24-1-0508 (M.-H.J.). The NIH supported the research reported in this publication. The content is solely the responsibility of the authors and does not represent the official view of the NIH.

## Author contributions

Y.S. and D.L. designed and conceived the study. Y.S., S.H.K., S.H.L., H.-c.T., M.E.B., and W.-C.T. performed the assays and manuscript writing. The other authors assisted with experiments and manuscript writing. All authors contributed to the article and approved the submitted version.

## Declaration of interests

No conflict of interest among the authors can be disclosed.

## Supplemental Information

Document S1. Figures S1–S6

Document S2. Article plus supplemental information
