# Supplementary material for: CD147-CAR-NK cell therapy shows minimal toxicities in human CD147 transgenic mouse model with solid tumors
Source: Mol Ther Oncol. 2025 Feb 26;33(1):200957. doi: 10.1016/j.omton.2025.200957 (PMC11952776; doi:10.1016/j.omton.2025.200957)
Supplement: Document S1. Figures S1–S6 [file mmc1.pdf]

OMTON, Volume *33*

Supplemental information

CD147-CAR-NK cell therapy shows minimal toxicities in human CD147 transgenic mouse model with solid tumors

Youssef Sabha, Sang Hoon Kim, Hsiang-chi Tseng, Maeve Elizabeth Byrne, Wei-Chung Tsao, Sang Hoon Lee, Zhongren Zhou, Mi-Hyeon Jang, and Dongfang Liu

SUPPLEMENTAL DATA

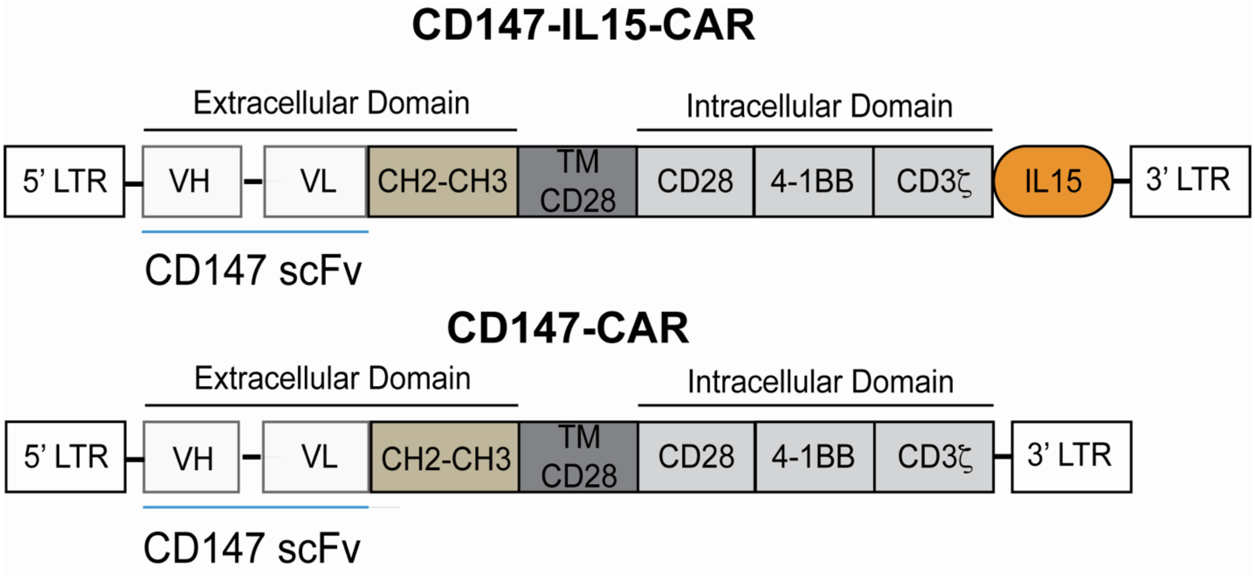

Figure S1: CD147-CAR and CD147-IL15-CAR Construct Design. Schematic outlining CAR structure and design. Both constructs contain a variable heavy and variable light chain targeting CD147, a human IgG1 CH2-CH3 domain, and a human CD28 transmembrane domain with a CD28-4-1BB-CD3ζ intracellular domain. The novel CD147-IL15-CAR construct contains an IL-15 domain downstream of the intracellular domain via 'self-cleaving' P2A (porcine teschovirus-1 2A) peptides (not shown in the diagram).

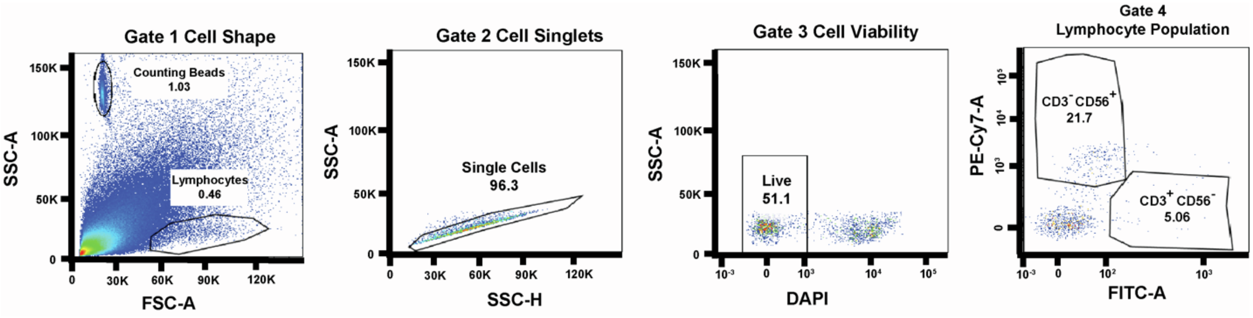

Figure S2: Tissue flow cytometry gating strategy for biodistribution experiment. Cells were gated on FSC/SSC lymphocyte populations, singlets that were viable based on DAPI staining. These cells were then gated on $CD3^{-}$ $CD56^{+}$ for NK Cells.

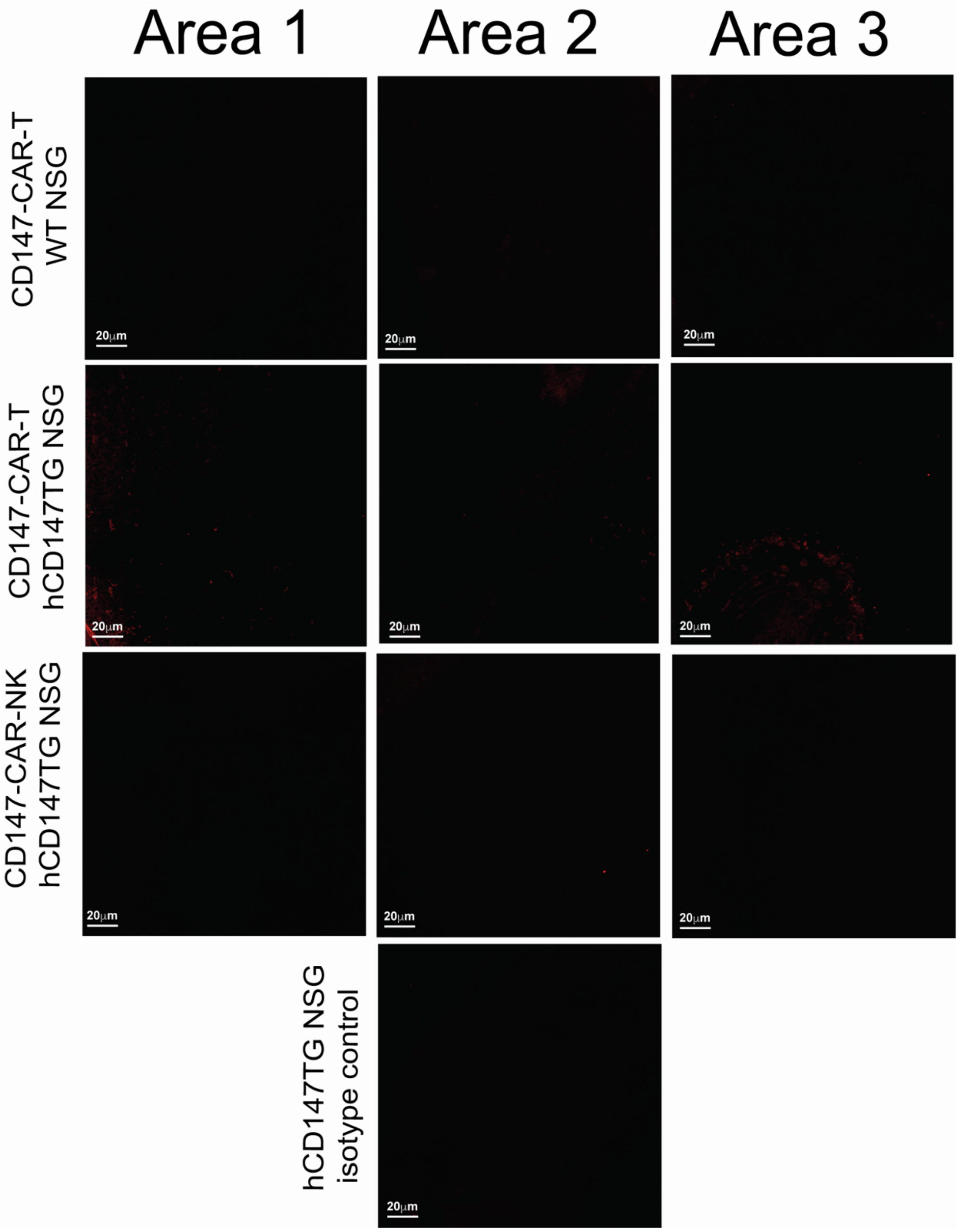

Figure S3: Representative confocal microscopy of INOS signal in brain tissues.

Representative mouse brain regions of interest regions after three doses of either CD147-CAR-T or CD147-CAR-NK. The CD147-CAR-T cells treated wild -type (WT) NSG mice were used as a negative control group. The red signal indicates INOS expression.

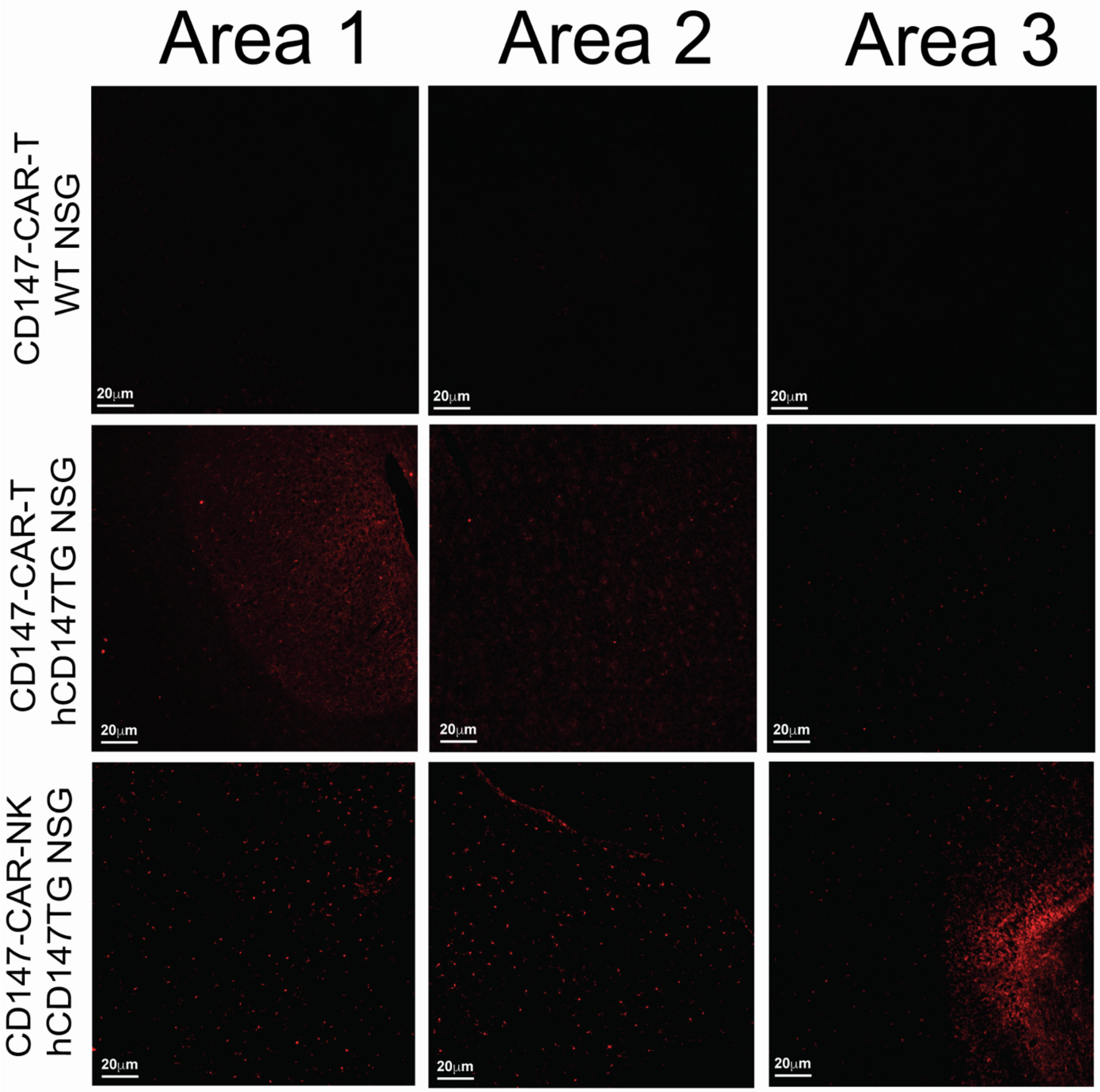

Figure S4: Representative confocal microscopy of IBA1 signal in brain tissues.

Representative mouse brain regions of interest regions after three doses of either CD147-CAR-T or CD147-CAR-NK. The CD147-CAR-T cells treated wild -type (WT) NSG mice were used as a negative control group. The red signal indicates IBA1 expressing tissue.

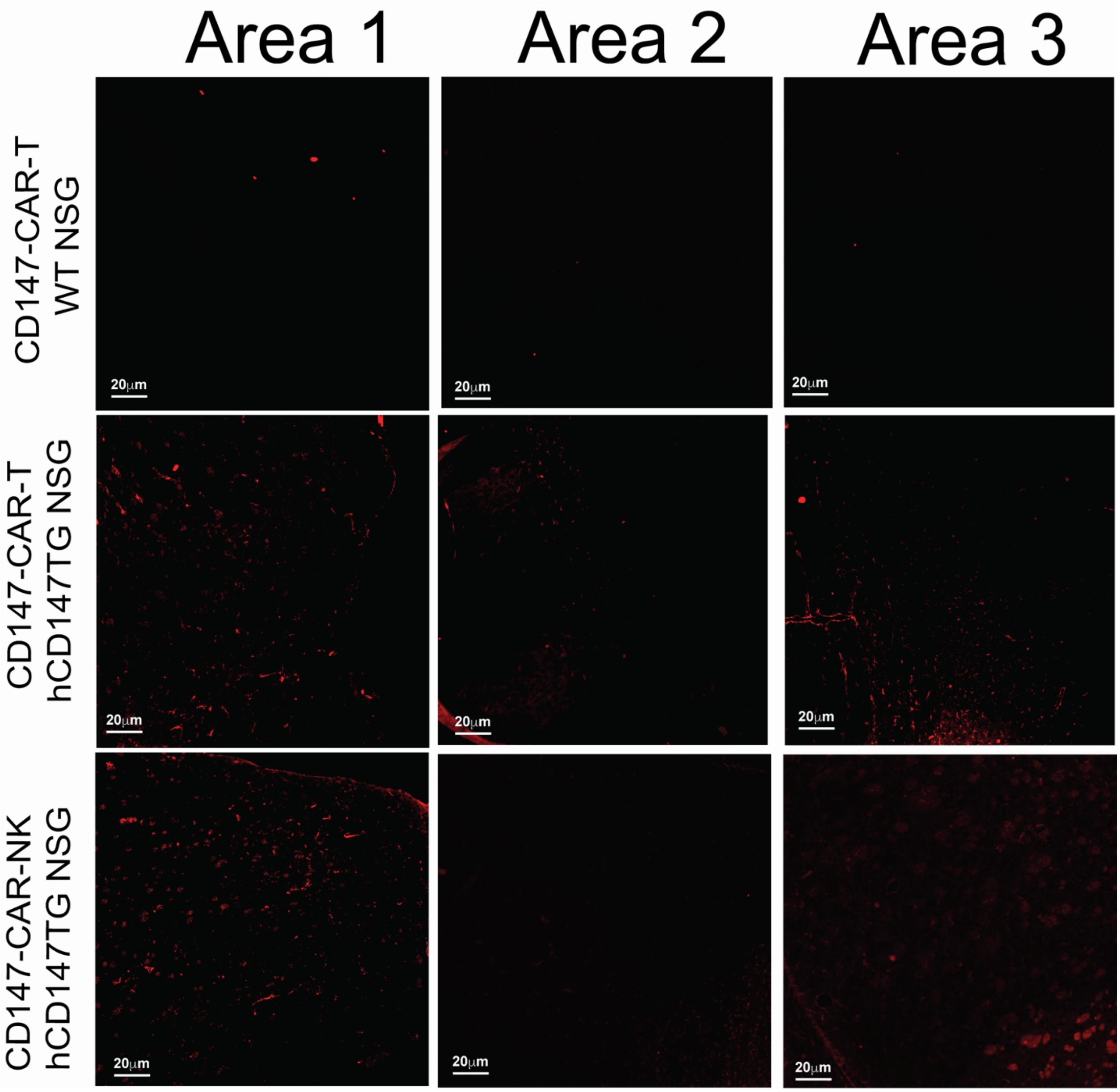

Figure S5. **Representative confocal microscopy of GFAP signal in brain tissues.**

Representative mouse brain regions of interest regions after three doses of either CD147-CAR-T or CD147-CAR-NK. The CD147-CAR-T cells treated wild -type (WT) NSG mice were used as a negative control group. The red signal indicates GFAP expressing tissue.

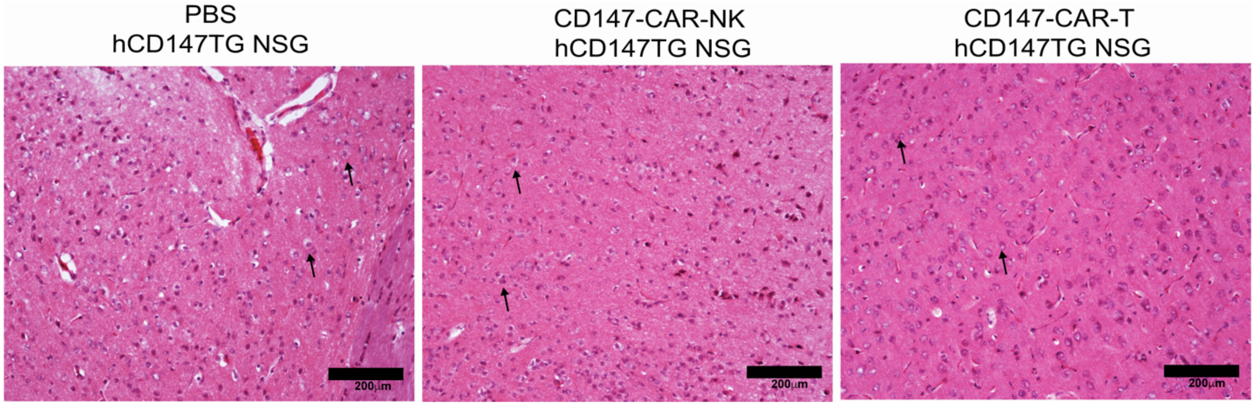

Figure S6. **Representative H&E staining of the brains from transgenic mice.**

Representative Hematoxylin and Eosin staining of brain sections from transgenic mice after 7 days of treatment with CAR therapy. PBS treated transgenic mouse brains were used as a negative control. Black arrows indicate immune cell populations.
